# FAM83A Promotes Lung Cancer Progression by Regulating the Wnt and Hippo Signaling Pathways and Indicates Poor Prognosis

**DOI:** 10.3389/fonc.2020.00180

**Published:** 2020-03-05

**Authors:** Yi-Wen Zheng, Zhi-Han Li, Lei Lei, Chen-Chen Liu, Zhao Wang, Liang-Ru Fei, Mai-Qing Yang, Wen-Jing Huang, Hong-Tao Xu

**Affiliations:** ^1^Department of Pathology, The First Hospital and College of Basic Medical Sciences, China Medical University, Shenyang, China; ^2^Department of Pathology, General Hospital of Heilongjiang Land Reclamation Bureau, Harbin, China; ^3^Department of Pathology, Changyi People's Hospital, Changyi, China

**Keywords:** FAM83A, lung cancer, Wnt signaling pathway, epithelial–mesenchymal transition, Hippo signaling pathway, proliferation, invasion

## Abstract

FAM83A (family with sequence similarity 83, member A) has been found to be highly expressed in cancers. The purpose of this study was to clarify the role and mechanism of FAM83A in lung cancers. The expression of FAM83A in lung cancer cells was enhanced by gene transfection or knocked down by small interfering RNA interference. The key proteins of the Wnt signaling pathway, the Hippo signaling pathway, and epithelial–mesenchymal transition (EMT) were examined using Western blot. The proliferation and invasion of lung cancer cells were examined using cell proliferation, colony formation, and invasion assays. The expression of FAM83A in lung cancer tissues was significantly increased and was correlated with advanced tumor–node–metastasis (TNM) stage and poor prognosis. Overexpression of FAM83A enhanced the proliferation, colony formation, and invasion of lung cancer cells. Meanwhile, FAM83A overexpression increased the expression of active β-catenin and Wnt target genes and the activity of EMT. Furthermore, in FAM83A-overexpressed cells, the activity of Hippo pathway was downregulated, whereas the expression of yes-associated protein (YAP) and its downstream targets cyclin E and CTGF were upregulated. The inhibitor of the Wnt signaling pathway, XAV-939, reversed the promoting effect of FAM83A on YAP, cyclin E, and CTGF. On knocking down the expression of FAM83A, we obtained the opposite results. However, the inhibitor of GSK3β, CHIR-99021, restored the expression of YAP, cyclin E, and CTGF after FAM83A was knocked down. FAM83A is highly expressed in lung cancers and correlated with advanced TNM stage and poor prognosis. FAM83A promotes the proliferation and invasion of lung cancer cells by regulating the Wnt and Hippo signaling pathways and EMT process.

## Introduction

Lung cancer is one of the malignancies exhibiting the fastest morbidity and mortality and the greatest threat to human health and life. Non–small cell lung cancer (NSCLC) accounts for 85% of new lung cancer cases (1), and 90% of patients with lung cancer die of complications related to tumor metastasis (2). Therefore, it is of great significance to study the mechanism of the occurrence and development of lung cancer and the proliferation, invasion, and metastasis of tumors to identify new therapeutic targets and approaches for the prevention and treatment of lung cancer.

Family with sequence similarity 83, member A (FAM83A), also known as bj-tsa-9, is located on chromosome 8q24 and was initially identified as a potential tumor-specific gene using a bioinformatics method (3). It consists of 434 amino acids, including DUF1669, serine-rich, and protein-rich domains (4), and the DUF1669 domain at its N-terminus is thought to be involved in tumor progression (5, 6). It has been reported that FAM83A is overexpressed in a variety of human tumors including lung, breast, testicular, and bladder cancers, suggesting that FAM83A may play a carcinogenic role in the development of cancer (7–9). Moreover, FAM83A is associated with poor prognosis (10). FAM83A is located downstream of the EGFR and PI3K pathways and correlated with the RAS/RAF/MEK/ERK and PI3K/AKT/mTOR pathways (4). Meanwhile, silencing FAM83A significantly reduces the ability of breast cancer cells to proliferate *in vitro* and *in vivo* and to grow and invade independently of anchoring (4). In addition, the use of 3D phenotypic regression analysis has shown that FAM83A in breast cancer may lead to resistance to tyrosine kinase inhibitors by EGFR/PI3K/AKT signaling pathway activation via c-RAF and PI3K p85 interactions, suggesting that excessive FAM83A expression may lead to drug resistance (11). At present, the role of FAM83A in the development of lung cancer is not clear, and its potential underlying mechanism also needs to be clarified (12).

In this study, we explored the regulatory effect of FAM83A on the Wnt and Hippo signaling pathways and epithelial–mesenchymal transition (EMT) by increasing or decreasing the expression of FAM83A in lung cancer cells and examined the effects of FAM83A on the proliferation and invasion of lung cancer cells.

## Materials and Methods

### Cell Culture and Transfection

Human lung cancer cell lines A549 and H1299 were purchased from the American Type Culture Collection (Manassas, VA, USA). Cell culture was performed in RPMI-1640 (Gibco, Invitrogen, Grand Island, NY, USA), and 10% fetal bovine serum (FBS; Gibco, Invitrogen) was added at 37°C in 5% CO_2_. The cells were grown in sterile culture dishes and passaged with 0.25% trypsin (Gibco, Invitrogen) every 1 or 2 days.

For transfection, cells were seeded in six-well plates 24 h before the experiment. The pCMV6–FAM83A plasmid and control empty vector pCMV6 were purchased from Origene (Rockville, MD, USA). Small interfering (Si) RNA against FAM83A (FAM83A-SiRNA) and control SiRNA were purchased from RiboBio (Guangzhou, China). According to the manufacturer's instructions, plasmids or SiRNAs were transfected into cells using Lipofectamine® 3000 (Invitrogen, Carlsbad, CA, USA) (13).

The inhibitor of Wnt/β-catenin signaling XAV-939 and GSK-3α/β inhibitor CHIR-99021 were purchased from MCE (MedChemExpress, Monmouth Junction, NJ, USA). Twenty-four hours later, transfection inhibitors were added. Meanwhile, dimethyl sulfoxide (DMSO; Beijing Solarbio Science & Technology Co., Beijing, China) settings were added for comparison. The concentrations of XAV-939 and CHIR-99021 were 20 nM.

### Western Blot Analysis

Total protein from cells was extracted in cell lysis buffer (Pierce, Rockford, IL, USA) and quantified using the Bradford method (14).

A total of 40 or 60 μg of protein was separated using 10% sodium dodecyl sulfate polyacrylamide gel electrophoresis and then transferred to a polyvinylidene fluoride membrane (Millipore, Bedford, MA, USA). The membrane was blocked with 5% non-fat milk for 2 h and incubated overnight at 4°C with antibodies against FAM83A (1:700, Origene, #TA335330), cyclin D1 (1:100, #sc-8396; Santa Cruz Biotechnology, Santa Cruz, CA, USA), MMP7 (1:100, #sc-515703; Santa Cruz Biotechnology), GAPDH (1:2,000, #sc-47724; Santa Cruz Biotechnology), β-catenin (1:500, #8480; Cell Signaling Technology, Danvers, MA, USA), active β-catenin (1:500, #8814; Cell Signaling Technology), E-cadherin (1:500, #3195; Cell Signaling Technology), GSK3β (1:500, #9832; Cell Signaling Technology), phosphorylated yes-associated protein (p-YAP) (1:500, #13008; Cell Signaling Technology), cyclin E (1:500, #11554-1-AP; Proteintech, Chicago, IL, USA), Twist (1:500, #25465-1-AP; Proteintech), Snail (1:500, #13099-1-AP; Proteintech), vimentin (1:500, #10366-1-AP; Proteintech), YAP (1:500, #13584-1-AP; Proteintech), phosphorylated GSK3β (p-GSK3β) (1:500, #14850-1-AP; Proteintech), LATS1 (1:500, #17049-1-AP; Proteintech), MST1 (1:500, #22245-1-AP; Proteintech), CTGF (1:500, #23936-1-AP; Proteintech), and c-Myc (1:500, #551101; BD Biosciences, San Jose, CA, USA). After washing, the membranes were incubated with horseradish peroxidase–conjugated mouse/rabbit immunoglobulin G (1:2,000; Proteintech) at 37°C for 2 h. Protein bands were visualized using electrochemiluminescence substrate (Pierce) and detected by using BioImaging Systems (DNR, Jerusalem, Israel). GAPDH protein levels were used as the control group to calculate relative protein levels.

### Cell Proliferation Assay

Twenty-four hours after transfection, cells were plated in 96-well plates (~3,000 cells per well) in medium with 10% FBS. Cell Counting Kit-8 (Dojindo Molecular Technologies, Tokyo, Japan) was used to detect cell proliferation. Cell Counting Kit-8 reagent was added to each well at a 1:10 (vol/vol) dilution per 100 μL and incubated at 37°C for 2 h. Our results were quantified spectrophotometrically at a wavelength of 450 nm (13).

### Colony Formation Assay

Twenty-four hours after transfection, cells were plated in 6-cm cell culture dishes and incubated for 14 days. To observe the results better, the dishes of the transfection group were plated with ~500 cells per dish, and the dishes of the interference group were plated with ~1,000 cells per dish. The medium was changed every 4–5 days. The cells were fixed with 4% paraformaldehyde for 20 min and stained with hematoxylin for 10 min. Colonies >0.3 mm in diameter were counted and recorded (13).

### Matrigel™ Invasion Assay

Twenty-four hours after transfection, Transwell chambers (Costar, Cambridge, MA, USA) and Matrigel (BD Biosciences) were used to assess the invasive ability of transfected cells according to the manufacturers' instructions. In brief, 100 μL Matrigel (1:7 dilution) was added to each insert. We placed the chambers in an incubator at 37°C for at least 2 h. After gelation of the Matrigel, we added cells in 100 μL of medium supplemented with 2% FBS. Among them, to better observe the results, the upper chamber included ~6 × 10^4^ cells in the transfection group and ~10 × 10^4^ cells in the interference group. Inversely, the lower chamber included the medium supplemented with 20% FBS as a chemical attractant. After 20 h of incubation, the filters were fixed with 4% paraformaldehyde for 20 min and stained with hematoxylin for 10 min. The non-invading cells on the upper surface were removed by scrubbing with a cotton tip. Five high-power fields were randomly selected under the microscope to count the number of invasive cells. The experiments were performed in triplicate (13, 15).

### Statistical Analysis

The statistical package GraphPad Prism 6.0 software (La Jolla, CA, USA) was used for all analyses. All values are expressed as mean ± standard deviation, and all results were analyzed using the Student *t*-test. *P* < 0.05 was considered statistically significant.

## Results

### Expression of FAM83A Is Increased in Lung Cancers and Correlated With Poor Survival

**Figure 1:**
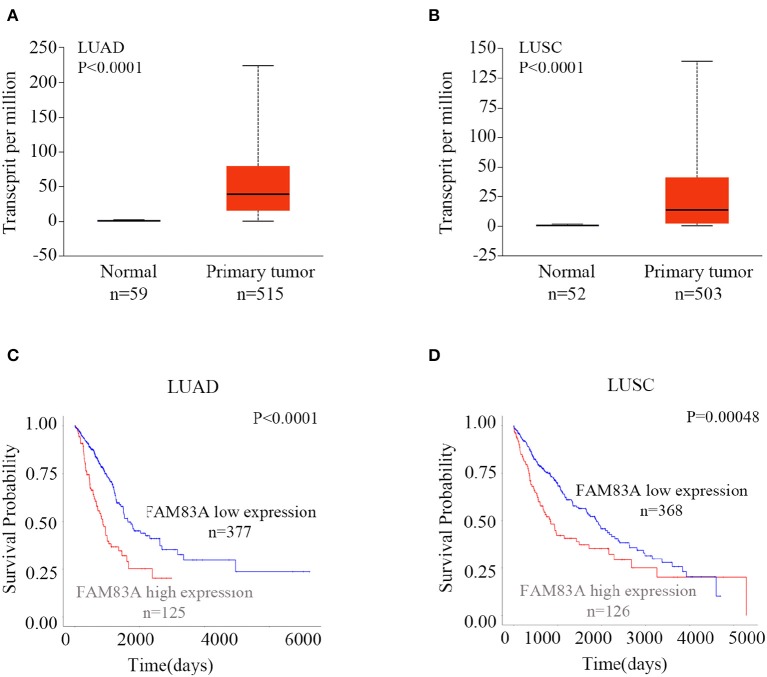
Expression of FAM83A in lung cancers and its correlation with prognosis. Expression levels of FAM83A in lung cancers and normal lung tissues, and their significant relation to the prognosis of patients with lung cancer. Box plots of FAM83A expression levels in lung adenocarcinomas (LUADs) **(A)** and lung squamous cell carcinomas (LUSC) **(B)** compared to normal lung tissues, which were retrieved from the UALCAN database. Kaplan–Meier curves of FAM83A expression in LUAD **(C)** and LUSC **(D)**, as retrieved from the UALCAN database.

To explore the expression and prognostic value of FAM83A, we retrieved the online database UALCAN (http://ualcan.path.uab.edu/index.html) (16). According to the database, the expression of FAM83A in both lung adenocarcinomas (LUADs) (*P* < 0.001) and lung squamous cell carcinomas (LUSCs) (*P* < 0.001) was significantly higher than that in normal lung tissues (Figures 1A,B). Both patients with LUAD and LUSC with high expression of FAM83A had shorter survival probability than that of patients with low levels of FAM83A expression (*P* < 0.001; Figures 1C,D). The online database, the Human Protein Atlas (https://www.proteinatlas.org/ENSG00000147689-FAM83A/pathology) (17), also showed that high expression of FAM83A was correlated with poor prognosis of patients with pancreatic cancer (*P* < 0.001; Figure S1A) and endometrial cancer (*P* < 0.001; Figure S1B). By analyzing the data downloaded from the cBioPortal database (http://www.cbioportal.org), we found that the mRNA level of FAM83A was positively correlated with the tumor (T) stage (correlation coefficient = 0.176, *P* < 0.001), lymph node (N) stage (correlation coefficient = 0.195, *P* < 0.001), metastasis (M) stage (correlation coefficient = 0.090, *P* = 0.041), and TNM stage (correlation coefficient = 0.231, *P* < 0.001) (Table S1) (18).

In addition, we also investigated the expression of FAM83B and FAM83C in lung cancers in the database. The expressions of FAM83B and FAM83C in both LUADs (*P* < 0.001) and LUSCs (*P* < 0.001) were significantly higher than that in normal lung tissues. However, only LUAD patients with high expression of FAM83B had shorter survival probability than that of patients with low levels of FAM83B expression (*P* < 0.001). High expression of FAM83C was not correlated with poor prognosis of patients with LUAD (*P* = 0.87) or LUSC (*P* = 0.052) (Figures S2, S3).

### FAM83A Promotes Proliferation and Colony Formation of Lung Cancer Cells

**Figure 2:**
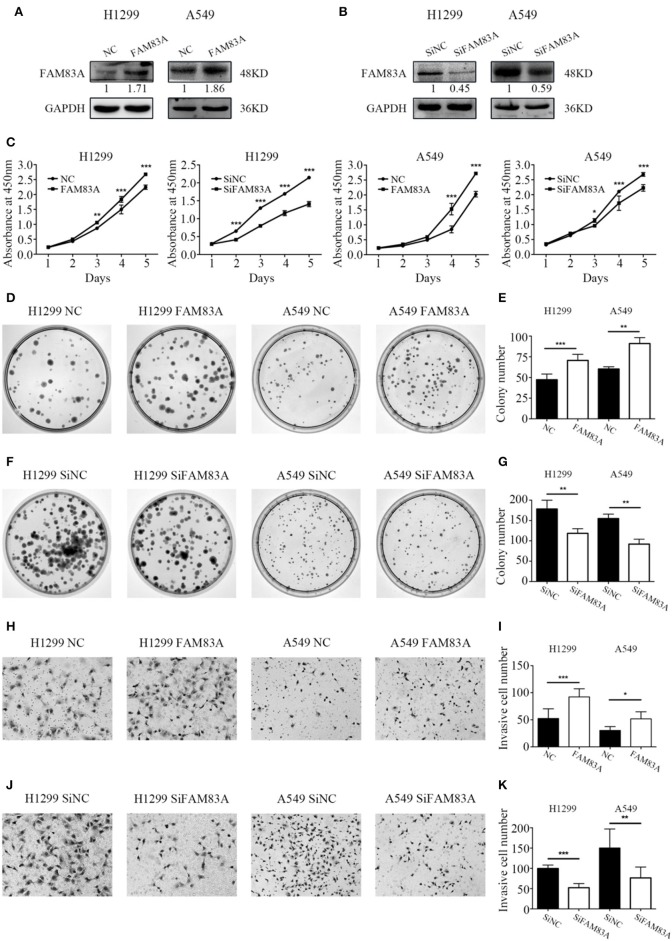
Effects of FAM83A on the proliferation, colony formation, and invasion of lung cancer cells. **(A,B)** The expression level of FAM83A of H1299 and A549 cells transfected with FAM83A or SiFAM83A. **(C)** The cell growth curve of H1299 and A549 cells transfected with FAM83A or SiFAM83A, and their control cells. **(D–G)** Representative images of the colony formation assay for H1299 and A549 cells transfected with FAM83A **(D)** or SiFAM83A **(F)**, and their control cells. The number of colonies formed by each group is shown in the histogram **(E,G)**. Representative images of the Matrigel invasion assay for H1299 and A549 cells transfected with FAM83A **(H)** or SiFAM83A **(J)** and their control cells. The invasive cell number for each group is shown in the histogram **(I,K)**. *P < 0.05, **P < 0.01, ***P < 0.001, NC, negative control cells; FAM83A, cells transfected with FAM83A; SiNC, cells interfered with control SiRNA; SiFAM83A, cells interfered with SiFAM83A.

The expression level of FAM83A was upregulated by FAM83A gene transfection in H1299 (H1299-FAM83A) and A549 (A549-FAM83A) cells (Figures 2A,B). Compared with those of control cells, the proliferation rate (H1299-FAM83A, *P* < 0.001; A549-FAM83A, *P* < 0.001) and number of colony formations (H1299-FAM83A, *P* < 0.001; A549-FAM83A, *P* = 0.0078) of lung cancer cells were significantly increased (Figures 2C–E). In contrast, when the expression of FAM83A was downregulated with SiRNA interference in H1299 (H1299-SiFAM83A) and A549 (A549-SiFAM83A) cells, the proliferation rate (H1299-SiFAM83A, *P* < 0.001; A549-SiFAM83A, *P* < 0.001) and number of colony formations (H1299-SiFAM83A, *P* = 0.0091; A549-SiFAM83A, *P* < 0.001) of lung cancer cells were significantly decreased compared with those of control cells (Figures 2C,F,G).

### FAM83A Promotes the Invasive Ability of Lung Cancer Cells

The Matrigel invasion assay showed that overexpression of FAM83A enhanced the invasive ability of H1299-FAM83A (*P* < 0.001) and A549-FAM83A (*P* = 0.012) cells compared to those of control cells (Figures 2H,I). Meanwhile, knocking down the expression of FAM83A inhibited the invasive ability of H1299-SiFAM83A (*P* < 0.001) and A549-SiFAM83A (*P* = 0.0016) cells compared to those of control cells (Figures 2J,K).

### FAM83A Enhances the Activity of the Wnt Signaling Pathway

**Figure 3:**
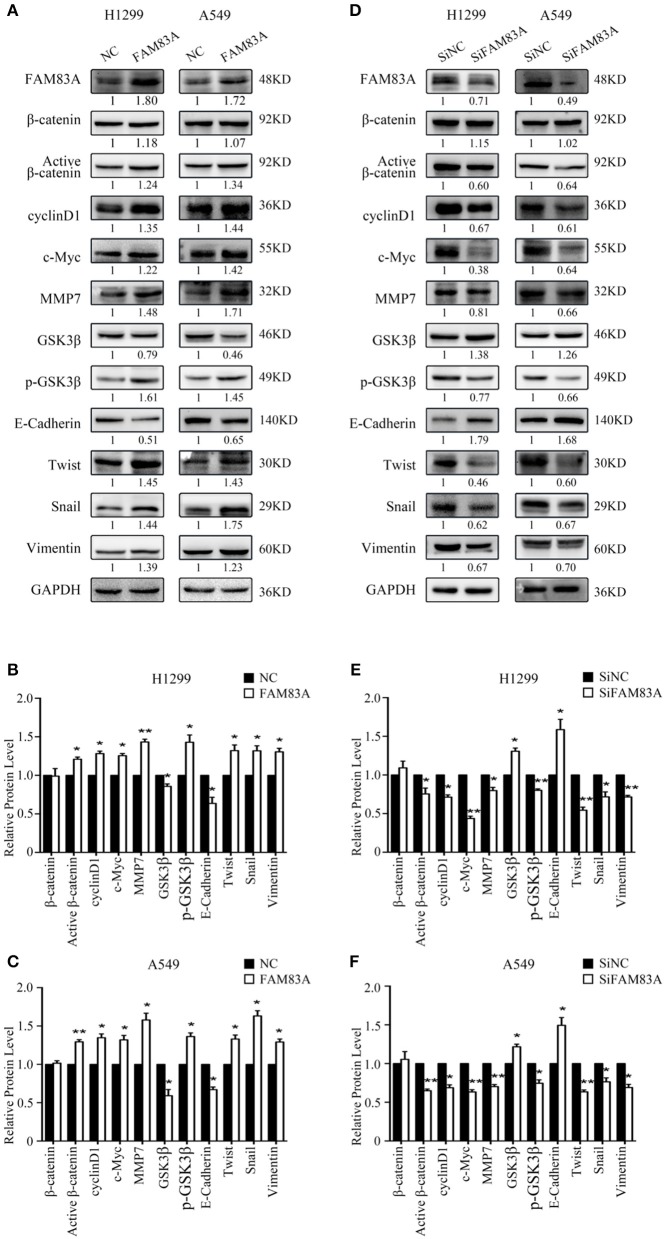
Expression of Wnt- and EMT-related genes under the regulation of FAM83A in lung cancer cells. Expression of Wnt signaling pathway- and EMT-related proteins after FAM83A overexpression **(A)** or knockdown **(D)** and relative protein levels in H1299 cells **(B,E)** and A549 cells **(C,F)**. GAPDH served as an internal control (*P < 0.05, **P < 0.01, NC, negative control cells; FAM83A, cells transfected with FAM83A; SiNC, cells interfered with control SiRNA; SiFAM83A, cells interfered with SiFAM83A).

We first detected the expression levels of FAM83A after transfection and SiRNA interference and then conducted the subsequent experiments. After FAM83A gene transfection, the expression level of total β-catenin, a key protein of the Wnt signaling pathway, was not altered, but the level of active β-catenin was significantly increased. Along with active β-catenin, the expression levels of cyclin D1, c-Myc, and MMP7, the target proteins of the Wnt pathway, were also upregulated. However, the level of GSK3β was decreased, whereas that of p-GSK3β was increased in H1299-FAM83A and A549-FAM83A cells (Figures 3A–C). Conversely, the expression levels of active β-catenin, p-GSK3β, cyclin D1, c-Myc, and MMP7 were downregulated, whereas that of GSK3β was upregulated in H1299-SiFAM83A and A549-SiFAM83A cells (Figures 3D–F).

### FAM83A Facilitates the EMT Process

In H1299-FAM83A and A549-FAM83A cells, the expressions of Twist and Snail, the EMT-inducing transcription factors, and vimentin, the mesenchymal marker, were all upregulated, whereas that of the cell adhesion molecule E-cadherin was downregulated (Figures 3A–C). On the contrary, in H1299-SiFAM83A and A549-SiFAM83A cells, the expressions of Twist, Snail, and vimentin were decreased, whereas the expression of E-cadherin was increased (Figures 3D–F).

### FAM83A Inhibits the Activation of the Hippo Signaling Pathway

**Figure 4:**
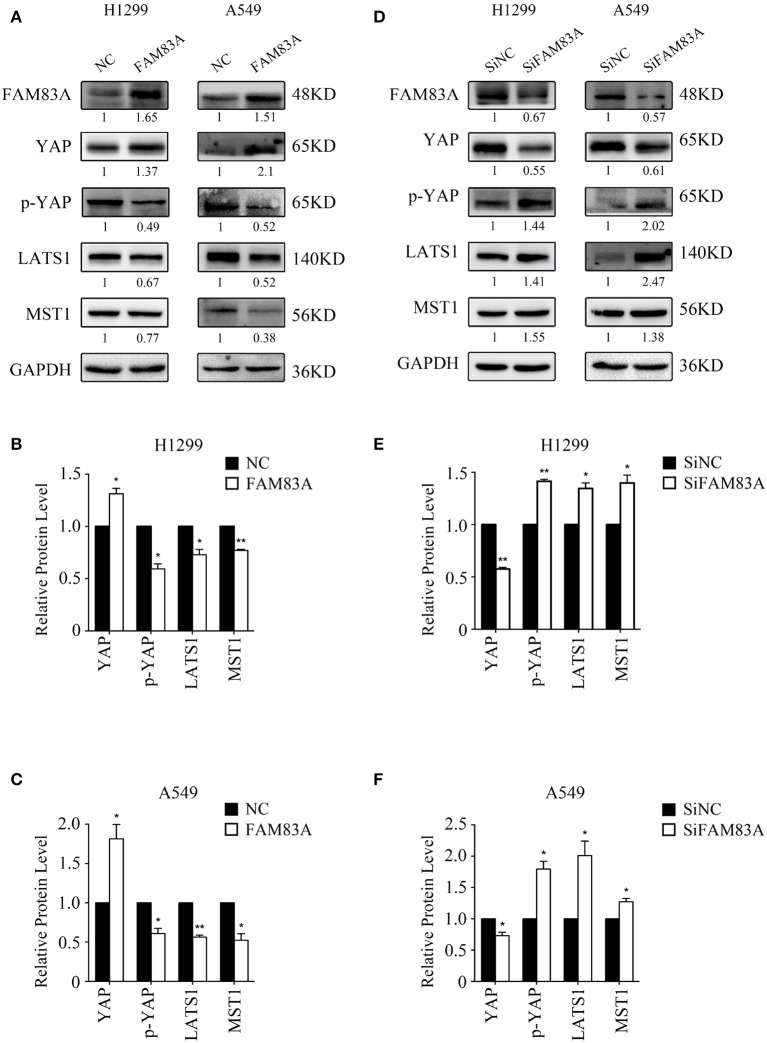
FAM83A inhibits the activation of the Hippo signaling pathway. Representative results of Western blot analysis **(A,D)** and relative protein levels in H1299 cells **(B,E)** and A549 cells **(C,F)**. GAPDH served as an internal control (*P < 0.05, **P < 0.01, NC, negative control cells; FAM83A, cells transfected with FAM83A; SiNC, cells interfered with control SiRNA; SiFAM83A, cells interfered with SiFAM83A).

The expression of YAP, the downstream effector molecule of the Hippo pathway, was upregulated in H1299-FAM83A and A549-FAM83A cells. Meanwhile, the expressions of p-YAP, LATS, and MST were downregulated (Figures 4A–C). In contrast, in H1299-SiFAM83A and A549-SiFAM83A cells, the expression of YAP was downregulated, and the expression of p-YAP, LATS, and MST were upregulated (Figures 4D–F).

### FAM83A Enhances the Proliferative and Invasive Abilities of Lung Cancer Cells by the Wnt Signaling Pathway

**Figure 5:**
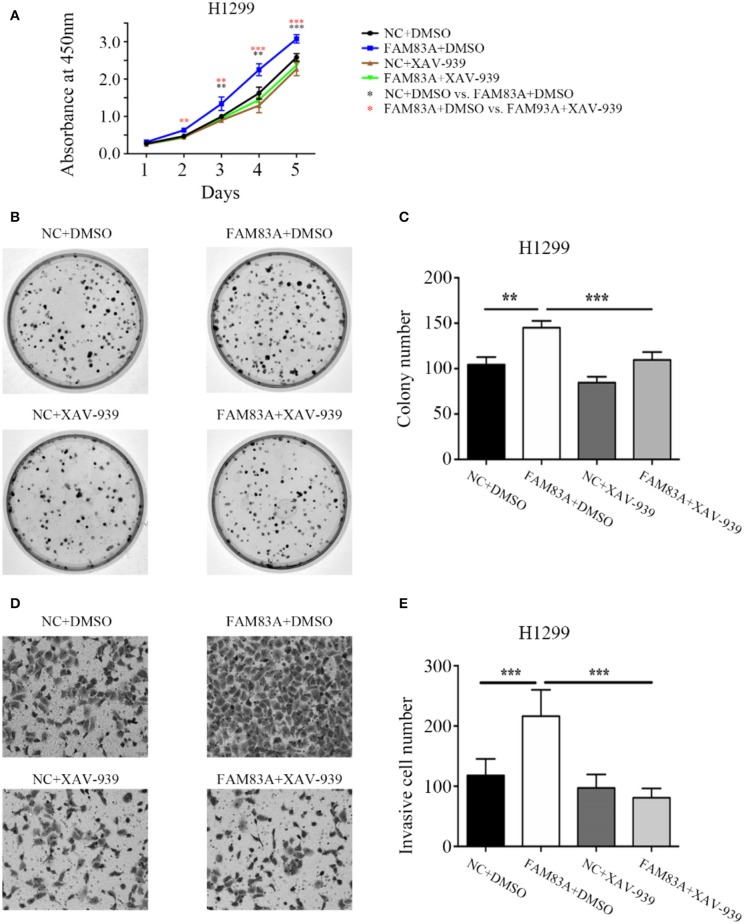
FAM83A enhances the proliferative and invasive abilities of lung cancer cells by the Wnt signaling pathway. **(A)** The cell growth curve of H1299 cells transfected with FAM83A, and their control cells were added with DMSO or XAV-939. **(B,C)** Representative images of the colony formation assay for H1299 cells transfected with FAM83A, and their control cells were added with DMSO or XAV-939 **(B)**. The number of colonies formed by each group is shown in the histogram **(C)**. **(D,E)** Representative images of the Matrigel invasion assay for H1299 cells transfected with FAM83A, and their control cells were added with DMSO or XAV-939 **(D)**. The invasive cell number for each group is shown in the histogram **(E)**. *P < 0.05, **P < 0.01, ***P < 0.001, NC, negative control cells; FAM83A, cells transfected with FAM83A; DMSO, dimethyl sulfoxide; XAV-939, an inhibitor of Wnt/β-catenin signaling; *NC+DMSO vs. FAM83A+DMSO; *FAM83A+DMSO vs. FAM83A+XAV-939.

To determine whether FAM83A promoted the proliferation and invasion of lung cancer cells through the Wnt signaling pathway, we added the Wnt inhibitor XAV-939 to H1299-FAM83A cells. Before the addition of Wnt inhibitor, we detected the transfection efficiency of FAM83A in H1299 by immunofluorescence assay. After ensuring a high transfection efficiency, the subsequent experiments were performed. Compared with H1299-FAM83A cells treated with DMSO, the proliferation rate (*P* < 0.001) and number of colony formations (*P* < 0.001) of H1299-FAM83A cells treated with XAV-939 were significantly decreased (Figures 5A–C). Similarly, the Matrigel invasion assay showed that H1299-FAM83A cells treated with XAV-939 had a reduced invasion capacity compared to the H1299-FAM83A with DMSO group (*P* < 0.001) (Figures 5D,E).

### GSK3β and the Wnt Signaling Pathway Contribute to FAM83A-Induced YAP Activation

**Figure 6:**
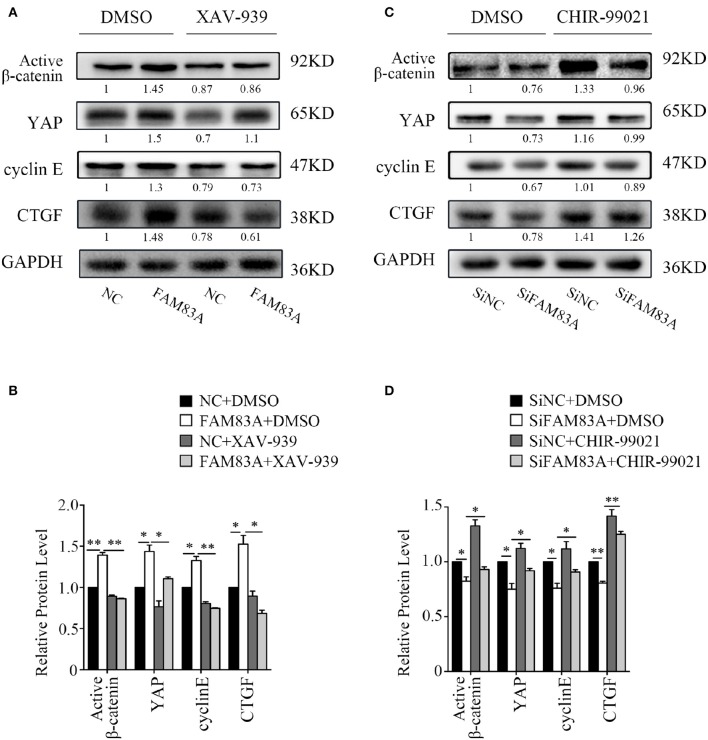
GSK3β and the Wnt signaling pathway contribute to FAM83A induced YAP activation. Expression of YAP and downstream proteins after FAM83A overexpression and adding Wnt inhibitor **(A)** or knocking down and adding GSK-3α/β inhibitor **(B)** in H1299 cells. Relative expression levels of proteins for each group are shown in the histogram **(C,D)**. *P < 0.05, **P < 0.01, NC, negative control cells; FAM83A, cells transfected with FAM83A; SiNC, cells interfered with control SiRNA; SiFAM83A, cells interfered with SiFAM83A; XAV-939, an inhibitor of Wnt/β-catenin signaling; CHIR-99021, a GSK-3α/β inhibitor.

To investigate the regulating mechanism of FAM83A on the Wnt and Hippo signaling pathways, we added XAV-939, an inhibitor of Wnt/β-catenin signaling, in H1299-FAM83A cells. The expression level of YAP was upregulated in H1299-FAM83A cells but was reversed when XAV-939 was added. The expressions of cyclin E and CTGF, the downstream targets of YAP, were upregulated in H1299-FAM83A cells but were also downregulated after the addition of XAV-939 (Figures 6A,B). We then used CHIR-99021, a GSK-3α/β inhibitor, in H1299-SiFAM83A cells for further investigation. The expression of YAP and its downstream targets cyclin E and CTGF were decreased in H1299-SiFAM83A cells but were restored after inhibiting the activity of GSK-3β using CHIR-99021 (Figures 6C,D).

## Discussion

FAM83A is involved in regulating a variety of different tumor-related signaling pathways, including the EGFR, RAS/RAF/MEK/ERK, and PI3K/AKT/mTOR pathways (3, 4). The overexpression of FAM83A has been shown in lung, breast, testicular, and bladder cancers and is associated with promoting the development of cancers (7–9, 19–22). Li et al. (3) reported that FAM83A is highly expressed in lung cancer tissues, especially LUAD. FAM83A was also found highly expressed in the peripheral blood of patients with lung cancer and had guiding significance in the patients prognosis and treatment of lung cancer. Lung Cancer Explorer database, which is containing lung cancer–specific expression data and clinical data from more than 6,700 patients in 56 studies, confirmed the association of FAM83A with poor prognosis in LUAD and LUSC (12). Richtmann et al. (10) also reported FAM83A has great potential as a diagnostic and prognostic marker of NSCLC and is closely related to tumor histology and EGFR expression and signal transduction. The present study confirmed the high expression of FAM83A in lung cancer. In addition, we also demonstrated that the high expression of FAM83A enhances the proliferative and invasive abilities of lung cancer cells and is associated with advanced TNM stage and poor prognosis of patients with lung cancer.

The mechanism of FAM83A-promoting lung cancer progression is not clear. To explore the underlying mechanism of FAM83A in lung cancer cells, we investigated the effects of FAM83A on the Wnt signaling pathway and EMT. We showed that FAM83A enhances the expression of active β-catenin and the target genes of the Wnt pathway, such as cyclin D1, c-Myc, and MMP7. Similar to our results, it has been reported previously that the Wnt signaling pathway is significantly inhibited after the silencing of FAM83A in pancreatic cancer cells (23). This suggests that FAM83A enhances the proliferative and invasive abilities of lung cancer cells by activating the Wnt signaling pathway. Meanwhile, as a target gene of the Wnt signaling pathway, the expression of Twist, an EMT-inducing transcription factor, is upregulated by the overexpression of FAM83A. Besides Twist, the levels of other EMT markers, such as Snail and vimentin, are also upregulated, whereas the level of epithelial marker E-cadherin is downregulated by FAM83A overexpression. During EMT, cell development changes from a polarized epithelial phenotype to a highly motile fibroblast or mesenchymal phenotype (24). EMT is involved in the early spread of cancer cells and plays an important role in the invasion and metastasis of tumors (25). Zhou et al. (26) reported that FAM83A induces EMT by the PI3K/AKT/Snail pathway in NSCLC. Thus, FAM83A facilitates the invasion and metastasis of lung cancer cells via promotion of the EMT process.

The expression of YAP, a downstream target of the Hippo signaling pathway, is increased in various tumors, such as those of lung, breast (27), colorectal (28), and oral cancers (29), and YAP/TAZ is carcinogenic. Yes-associated protein expression has been reported to be upregulated in 66.3% of NSCLC samples and is significantly correlated with NSCLC staging and lymph node metastasis (30). Therefore, we wondered about FAM83A's oncogenic role related to YAP and the Hippo signaling pathway. The main members of the Hippo pathway are MST1/2 kinase, LATS1/2 kinase, and their adaptor proteins Sav and MOB1 (31). When the Hippo signaling pathway is activated, MST1/2 binds to Sav and phosphorylates LATS1/2 and MOB1 to increase the LATS/MOB1 complex and activate LATS1/2. Active LATS1/2 phosphorylates and inactivates YAP (32). In this study, overexpression of FAM83A upregulated the level of YAP, but downregulated the expressions of MST, LATS, and p-YAP. This result indicates that FAM83A can inhibit the Hippo signaling pathway and enhance the activity of YAP.

How can FAM83A regulate YAP and the Hippo signaling pathway? It has been reported that YAP is also a target protein of the Wnt signaling pathway (33), and GSK3β is the key molecule connecting the Hippo pathway with the canonical Wnt/β-catenin pathway (34). Therefore, we investigated whether FAM83A regulates the Hippo pathway through GSK3β and the Wnt signaling pathway using specific inhibitors of GSK3β and the Wnt signaling pathway. XAV-939, an inhibitor of the Wnt signaling pathway, reverses the promoting effects of FAM83A overexpression on YAP and its downstream targets, cyclin E and CTGF. On the contrary, CHIR-99021, the inhibitor of GSK3β, restores the expression of YAP, cyclin E, and CTGF, which are downregulated in FAM83A knocked-down cells. These results suggest that FAM83A could regulate YAP expression and its downstream targets through GSK3β and the Wnt signaling pathway. GSK3β, as a key regulator for both the Wnt and Hippo signaling pathways, is not only an inhibitor of β-catenin, but can also regulate the activity of YAP. Thus, FAM83A may regulate the activities of both the Wnt and Hippo signaling pathways by inhibiting GSK3β, but the detailed mechanism still needs further investigation.

**Figure 7:**
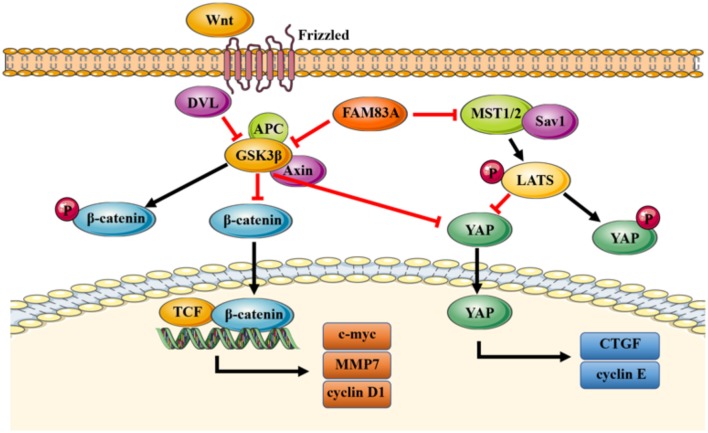
A proposed model to illustrate the role of FAM83A in the Wnt and Hippo signaling pathways. GSK3β phosphorylates β-catenin and results in the degradation of β-catenin, which inhibits the activity of the Wnt signaling pathway. FAM83A can inhibit GSK3β activity and increase the level of active unphosphorylated β-catenin; active β-catenin then transports into the nucleus and activates the Wnt signaling pathway. Meanwhile, similar to β-catenin, FAM83A may also inhibit the phosphorylation and degradation of YAP, which is induced by the Hippo signaling pathway and enhance the activity of YAP through repressing GSK3β. In addition, FAM83A could downregulate the upstream MST to inhibit the activation of the Hippo signaling pathway. Figures were produced using Servier Medical Art (https://smart.servier.com).

In conclusion, our study elucidates a novel mechanism of FAM83A inhibiting the Hippo signaling pathway through GSK3β and the Wnt signaling pathway (Figure 7). FAM83A is highly expressed in lung cancers and correlated with advanced TNM stage and poor prognosis. FAM83A promotes the proliferation and invasion of lung cancer cells by regulating the Wnt and Hippo signaling pathways and EMT process.

## Data Availability Statement

The datasets supporting the conclusions of this article are included within the article and its supplementary information files. FAM83A mRNA expression data of lung cancer patients were from UALCAN database (http://ualcan.path.uab.edu/index.html). Kaplan–Meier plots of overall survival of patients with lung cancer, pancreatic cancer and endometrial cancer stratified by FAM83A expression were obtained from the UALCAN (http://ualcan.path.uab.edu/index.html) and the Human Protein Atlas database (https://www.proteinatlas.org).

## Author Contributions

Y-WZ conceived the study, conducted experiments, acquired and analyzed data, and wrote the manuscript. ZW, C-CL, and LL provided suggestions and participated in data analysis. Z-HL and L-RF contributed to the collection of the tissue specimens. W-JH and M-QY contributed to data analysis. H-TX responsible for conception and supervision of the study, and wrote the manuscript. All authors corrected draft versions and approved the final version of the manuscript.

### Conflict of Interest

The authors declare that the research was conducted in the absence of any commercial or financial relationships that could be construed as a potential conflict of interest.

## Supplementary Material

Table S1 The correlation between FAM83A and the clinicopathological factors of lung adenocarcinomas. ^*^*P* < 0.05, ^**^*P* < 0.01. FAM83A, Family with sequence similarity 83, member A.

Figure S1 The expression level of FAM83A was associated with poor prognosis in pancreatic cancer and endometrial cancer. Kaplan–Meier curves of FAM83A expression in pancreatic cancer **(A)** and endometrial cancer **(B)**, as retrieved from the Human Protein Atlas database.

Figure S2 Expression of FAM83B in lung cancers and its correlation with prognosis. Expression levels of FAM83B in lung cancers and normal lung tissues, and their significant relation to the prognosis of patients with lung cancer. **(A,B)** Box plots of FAM83B expression levels in LUAD **(A)** and LUSC **(B)** compared to normal lung tissues, which were retrieved from the UALCAN database. Kaplan–Meier curves of FAM83B expression in LUAD **(C)** and LUSC **(D)**, as retrieved from the UALCAN database.

Figure S3 Expression of FAM83C in lung cancers and its correlation with prognosis. Expression levels of FAM83C in lung cancers and normal lung tissues, and their significant relation to the prognosis of patients with lung cancer. **(A,B)** Box plots of FAM83C expression levels in LUAD **(A)** and LUSC **(B)** compared to normal lung tissues, which were retrieved from the UALCAN database. Kaplan–Meier curves of FAM83C expression in LUAD **(C)** and LUSC **(D)**, as retrieved from the UALCAN database.

## Abbreviations

FAM83A: family with sequence similarity 83, member A
YAP: yes-associated protein
EMT: epithelial–mesenchymal transformation
p-GSK3β: phosphorylated GSK3β
p-YAP: phosphorylated YAP
LUAD: lung adenocarcinoma
LUSC: lung squamous cell carcinoma
TNM: tumor–node–metastasis
SiRNA: small interfering RNA.
