# Supplementary figures and images for: FAM83A Promotes Lung Cancer Progression by Regulating the Wnt and Hippo Signaling Pathways and Indicates Poor Prognosis
Source: Front Oncol. 2020 Mar 5;10:180. doi: 10.3389/fonc.2020.00180 (PMC7066079; doi:10.3389/fonc.2020.00180)

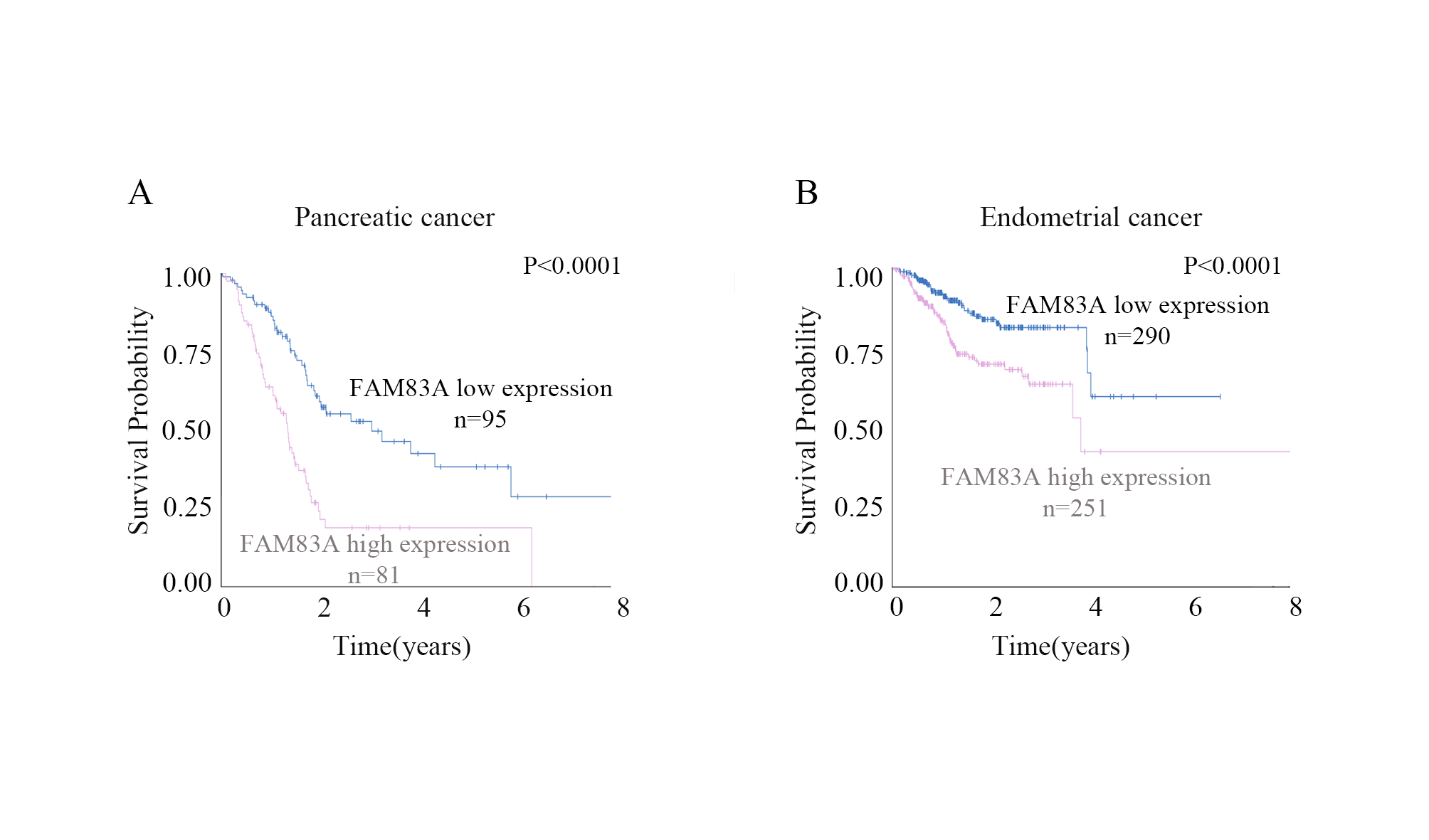

Supplement: Figure S1 — The expression level of FAM83A was associated with poor prognosis in pancreatic cancer and endometrial cancer. Kaplan–Meier curves of FAM83A expression in pancreatic cancer (A) and endometrial cancer (B), as retrieved from the Human Protein Atlas database. [file Image_1.TIF]

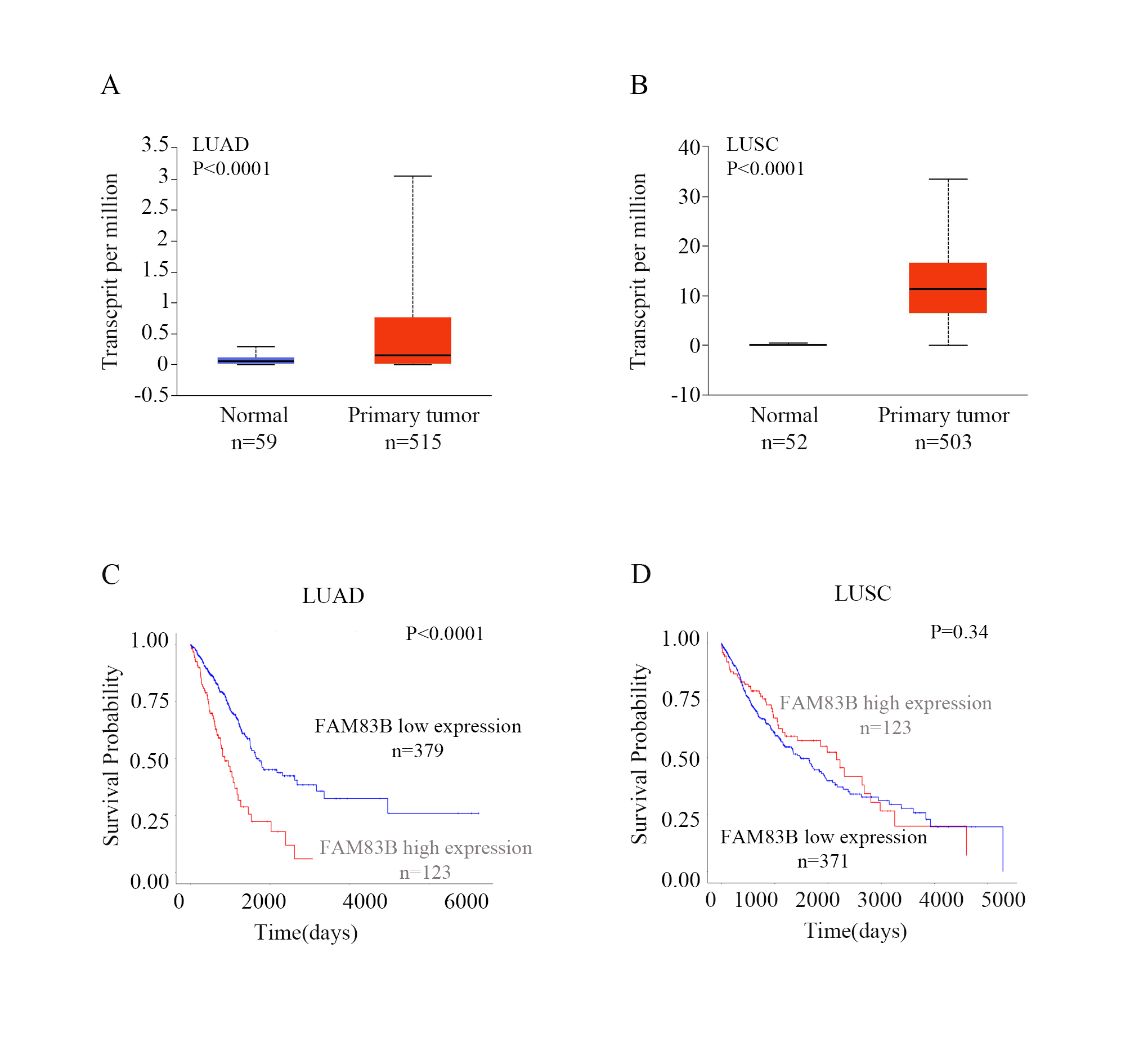

Supplement: Figure S2 — Expression of FAM83B in lung cancers and its correlation with prognosis. Expression levels of FAM83B in lung cancers and normal lung tissues, and their significant relation to the prognosis of patients with lung cancer. (A,B) Box plots of FAM83B expression levels in LUAD (A) and LUSC (B) compared to normal lung tissues, which were retrieved from the UALCAN database. Kaplan–Meier curves of FAM83B expression in LUAD (C) and LUSC (D), as retrieved from the UALCAN database. [file Image_2.TIF]

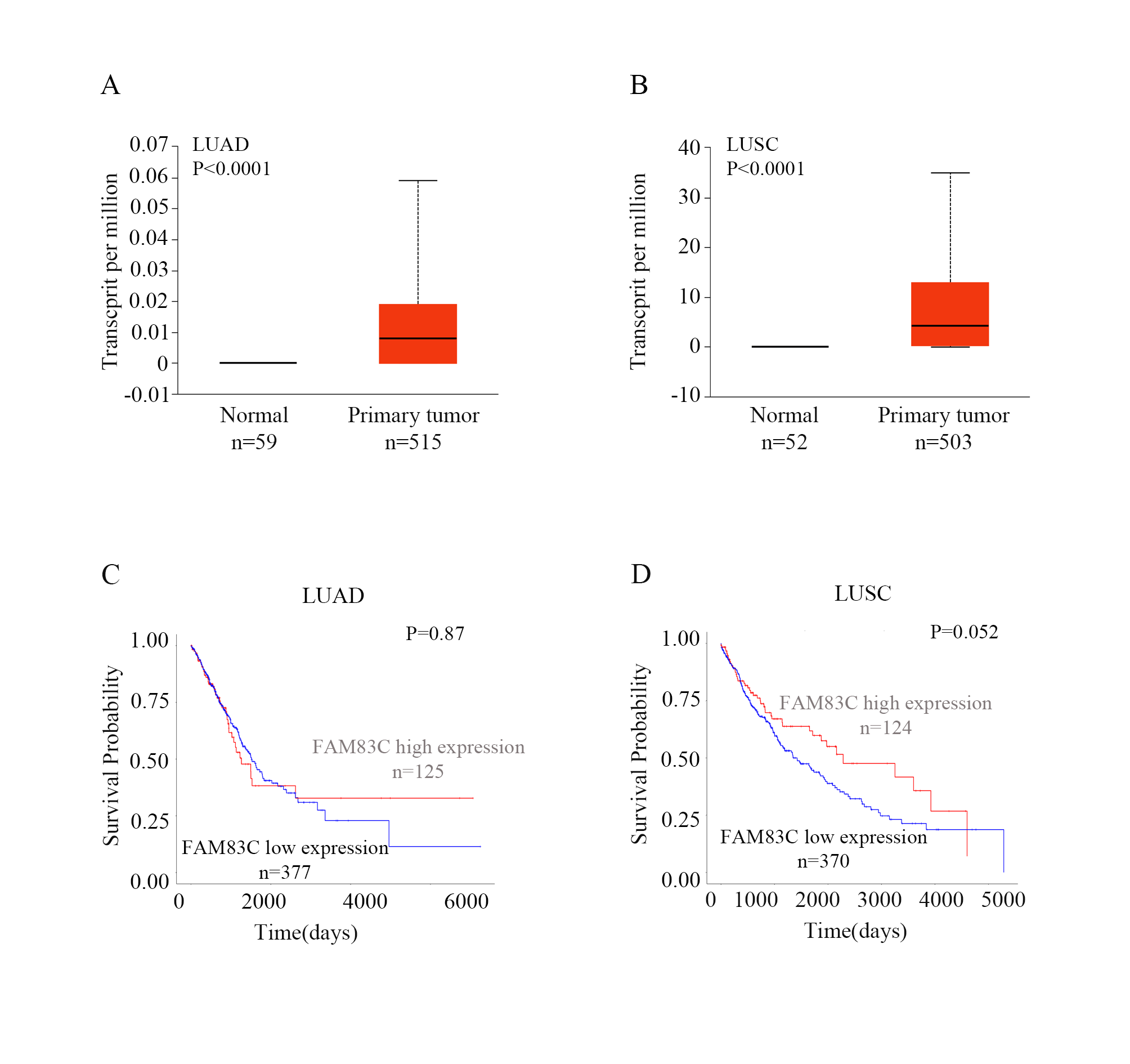

Supplement: Figure S3 — Expression of FAM83C in lung cancers and its correlation with prognosis. Expression levels of FAM83C in lung cancers and normal lung tissues, and their significant relation to the prognosis of patients with lung cancer. (A,B) Box plots of FAM83C expression levels in LUAD (A) and LUSC (B) compared to normal lung tissues, which were retrieved from the UALCAN database. Kaplan–Meier curves of FAM83C expression in LUAD (C) and LUSC (D), as retrieved from the UALCAN database. [file Image_3.TIF]
